# The Impact of Transformational Leadership on Safety Climate and Individual Safety Behavior on Construction Sites

**DOI:** 10.3390/ijerph14010045

**Published:** 2017-01-05

**Authors:** Yuzhong Shen, Chuanjing Ju, Tas Yong Koh, Steve Rowlinson, Adrian J. Bridge

**Affiliations:** 1 Department of Real Estate and Construction, The University of Hong Kong, Pokfulam Road, Hong Kong, China; tasykoh@hku.hk (T.Y.K.); steverowlinson@hku.hk (S.R.); 2 College of Civil Engineering, Shanghai Normal University, Shanghai 200234, China; 3 School of Economics and Management, Southeast University, Nanjing 211189, China; juchuanjing@outlook.com; 4 Science and Engineering Faculty, Queensland University of Technology, Brisbane, QLD 4001, Australia; a.bridge@qut.edu.au

**Keywords:** transformational leadership, safety climate, safety behavior, construction personnel, random sample

## Abstract

Unsafe acts contribute dominantly to construction accidents, and increasing safety behavior is essential to reduce accidents. Previous research conceptualized safety behavior as an interaction between proximal individual differences (safety knowledge and safety motivation) and distal contextual factors (leadership and safety climate). However, relatively little empirical research has examined this conceptualization in the construction sector. Given the cultural background of the sample, this study makes a slight modification to the conceptualization and views transformational leadership as an antecedent of safety climate. Accordingly, this study establishes a multiple mediator model showing the mechanisms through which transformational leadership translates into safety behavior. The multiple mediator model is estimated by the structural equation modeling (SEM) technique, using individual questionnaire responses from a random sample of construction personnel based in Hong Kong. As hypothesized, transformational leadership has a significant impact on safety climate which is mediated by safety-specific leader–member exchange (LMX), and safety climate in turn impacts safety behavior through safety knowledge. The results suggest that future safety climate interventions should be more effective if supervisors exhibit transformational leadership, encourage construction personnel to voice safety concerns without fear of retaliation, and repeatedly remind them about safety on the job.

## 1. Introduction

Due to substantial efforts from stakeholders, the accident and fatality rate in the Hong Kong construction industry have witnessed a continuous reduction in recent years. Throughout the years 2003–2012, the number of accidents per 1000 workers decreased from 68.1 to 44.3, and that of fatalities decreased from 0.39 to 0.34 [1]. However, there seems to be no reduction in the contribution of at-risk behaviors to accidents, evidencing that human actions are still the dominant cause of industrial accidents [2,3]. For example, in the public construction works, unsafe actions (including lack of attention, unsafe position or posture, and use of unsuitable access/failure to use access) appear in respectively 61%, 58%, 60%, and 54% of the total number of accidents during the years 2009–2012 [4].

Given these statistics, a considerable amount of research on safety behavior has been carried out. However, much occupational safety behavior research tends to “focus on either individual differences or contextual factors but rarely on both” [5] (p. 1103). In light of this knowledge gap, based on work performance and climate theories, Christian et al. [5] reviewed the safety literature in a meta-analysis of 90 studies to examine person- and situation-based antecedents of safety behavior and outcomes. In the meta-analysis, they established and estimated a conceptual framework depicting the impact of distal situation- and person-related factors on safety behavior and outcomes via proximal person-related factors. They categorized safety climate and leadership as distal situation-related antecedents, and safety motivation and knowledge as proximal person-related determinants. Christian et al.’s [5] conceptualization of safety behavior as an interaction between proximal individual differences (e.g., safety knowledge and safety motivation) and distal contextual factors (e.g., leadership and safety climate) is in line with contemporary construction accident causation models [6,7], which adopt a systems perspective and attribute incidents to a complex interaction between proximal factors (e.g., unsafe conditions and actions) and distal factors (e.g., management commitment and supervisor support).

In Christian et al.’s [5] model, safety climate and leadership (including leader–member exchange, LMX) are categorized in parallel as distal situation-related factors. This study further modifies the model and takes leadership as an antecedent of safety climate and safety-specific LMX as a mediator variable. The reasons are twofold. First, leaders at different hierarchical levels determine organizational climate, including safety climate. This is because from leaders’ response subordinates recognize which policies, procedures, practices and behaviors would be rewarded or supported [8]. Second, safety behavior is influenced by an individual’s cultural background. The sample of this study was mainly the Hong Kong Chinese, who are in a society higher in power distance. In such a society, leadership may have a significant impact on subordinates’ safety climate perceptions [8]. Therefore, after model modification this study aims to examine the mechanisms through which transformational leadership impacts safety behavior via safety climate.

The remainder of this paper unfolds as follows. It first addresses relationships between safety climate and safety behavior, and between leadership and safety climate, respectively. Then it proposes a hypothetical model relating transformational leadership to safety behavior through safety climate. Using data from a questionnaire survey of a random sample of construction personnel based in Hong Kong, it then estimates the hypothetical model and compares competing structural models. Finally, both theoretical and practical implications of the findings are discussed, along with limitations and future research directions.

## 2. Model Development

### 2.1. Impact of Safety Climate on Safety Behavior

The safety climate theory has its roots in social psychology, in recognition that behavior is derived from an interaction between the person and the psychological environment. Hence, the safety climate construct is proposed to understand and explain individuals’ safety behavior. In fact, the association between a positive and strong safety climate and safety behavior has been found across a variety of industrial sectors [5,8]. A meta-analysis of 32 studies by Clarke [9] confirms the positive relationship between safety climate and safety behavior in prospective studies, in which safety behavior is assessed following the measurement of safety climate. Among others, in construction a positive link is also reported between safety climate and safety performance, including safety behavior [10,11,12,13,14,15,16,17].

Safety climate has both direct and indirect influence on safety behavior. In a meta-analysis of 51 studies, Clarke [18] finds that the effects of safety climate on safety behavior are partially mediated by work attitudes (i.e., organizational commitment and job satisfaction). In explaining why unsafe work behavior is rampant in agriculture, Seo [19] finds that perceived safety climate affects at-risk work behavior through either the sequential influence of mediators (i.e., perceived work pressure, perceived risk, and perceived barriers), the sole mediator of perceived barriers, or a direct influence on at-risk behavior. Neal et al. [20] and Christian et al. [5] find that the effect of safety climate on safety behavior is partially mediated by safety knowledge and motivation.

Like Neal et al. [20] and Christian et al. [5], this study also examines the role of safety motivation and safety knowledge as two mediators in the relationship between safety climate and safety behavior (see Hypothesis 2) for three reasons. First, available evidence does support that safety knowledge and motivation are important determinants of safety behavior [20]. Drawing on Campbell’s et al. [21] work performance theory, Griffin and Neal [22] identify three safety behavior determinants: safety knowledge, safety skills, and safety motivation. Due to the difficulty in measuring safety skills, Neal et al. [20] do not consider the safety skills construct in their model. Furthermore, Griffin and Neal [22] identify safety behavior antecedents at both the individual level (e.g., ability, experience, and conscientiousness) and the organization level (e.g., safety climate). Specifically, they postulate that safety motivation and safety knowledge mediate the impact of safety climate on safety behavior. Second, safety climate reflects the extent to which employees believe that safety is valued in the organization. Based on these perceptions and feelings, employees develop expectations about behavior-outcome contingencies and accordingly adapt their behaviors [23,24,25]. In other words, safety climate determines safety motivation and hence safety behavior. Third, safety climate influences safety knowledge as well. In a sound and positive safety climate, employees’ safety knowledge would increase through more communication about safety matters with colleagues. For a safety climate to be effective, there must be a strong leadership and positive leader-subordinate relationships [26]. The following section addresses the impact of leadership on safety climate.

### 2.2. Impact of Transformational Leadership on Safety Climate

Leadership concerns a process of influencing, through which the leader increases the followers’ acceptance of objectives and achieving methods, and facilitates their individual and collective efforts towards the shared objectives [27]. Scholars identify two types of leadership styles, transformational and transactional leadership [28]. The transactional–transformational leadership paradigm is broad enough to measure and understand the leadership construct [25]. Transactional leadership emphasizes effective and efficient task organization and reliable task accomplishment, whereas transformational leadership focuses on inspiring the followers to transcend their own self-interests for the collective good through altering their need level on Maslow’s hierarchy. Transformational supervisors usually inspire their subordinates to transcend their personal interests with four influence tactics: serving as a role model for the desired behavior (idealized influence or charisma), developing subordinates’ goal commitment (inspirational appeals or motivation), intellectually engaging subordinates in problem solving (intellectual stimulation), and empathizing with subordinates (individualized consideration). Although there are many leadership styles, this paper chooses to examine the impact of transformational leadership on safety climate. This is because, transformational leaders foster closer relationships with subordinates [29], which is more fitting for the Hong Kong context where construction personnel are relationship-oriented [30].

Transformational leadership is essential in construction safety management. First, transformational leadership is essential in the construction process [8]. Continual changes feature the construction process [31], and addressing continual changes entails transformational leadership [32]. In a survey of 510 building professionals across Australia, Hong Kong, Singapore, and the U.K., Chan and Chan [33] find that leaders in construction use both transformational and transactional leadership styles. Furthermore, they report that all transformational leadership factors (i.e., influence tactics) and the transactional leadership factor of contingent reward correlate significantly with leadership outcomes (i.e., leader effectiveness, extra effort by employees, and employees’ satisfaction with the leader). Second, the critical role of transformational leadership in safety performance is well established. Treating safety as a work value may be an effective way to improve safety performance. In order to instill this value in employees, the entire management structure should proactively and visibly demonstrate its leadership of and commitment to safety on a daily basis [34]. The management should develop a vision that specifies the company’s objective, safety standards and required action. Furthermore, in order to sell the vision, the management must commit to the vision, “walk the talk”, and lead by example. In other words, the management must employ the influence tactics of transformational leadership to improve the company’s safety performance. Empirical research also corroborates this point. Safety knowledge and safety motivation are well-established predictors of safety participation, but what is less known is the impact of leadership styles on these relationships [35]. In a survey of employees from a large metropolitan public transit agency in the northwestern U.S., Jiang and Probst [35] find that transformational leadership strengthens the safety knowledge–participation relationship. Transformational leadership is associated with positive safety outcomes, such as improved safety climate, increased safety behaviors, and decreased accidents and injuries [36]. However, different facets of transformational leadership have different impacts on safety outcomes. In a survey of 1167 construction pipefitters and plumbers, Hoffmeister et al. [36] find that idealized influence account for the most variance in safety outcomes, whereas individualized consideration often accounts for the least amount of variance.

Leaders determine organizational climate. This is because from leaders’ response subordinates identify which policies, procedures, practices and behaviors would be rewarded or supported [8]. For transformational leadership to produce a positive safety climate, a mediator variable may be needed [8]. With a random sample of construction personnel based in Hong Kong, Shen et al. [8] find that a construct of safety-specific leader–member exchange mediates the impact of transformational leadership on safety climate (see Hypothesis 1). The safety-specific leader–member exchange refers to interactions between employees and their immediate supervisors on safety issues. Project activities are carried out through the collective interactions of project participants [37]. Construction personnel interact with their immediate supervisors on a daily basis. Therefore, it is reasonable to suspect that construction personnel are more likely to believe that safety is valued when their immediate supervisors take safety seriously in their daily interactions.

### 2.3. Safety Behavior

In general, the term safety performance may refer to two different concepts [5]. It may refer to an organizational metric for safety outcomes, such as number of accidents per 1000 workers. Alternatively, it may refer to a metric for individuals’ safety behaviors. The former is the consequence of accidents, while the latter predicts accidents. Recent years have seen a shift in safety measurement from lagging indicators to leading ones. Therefore, this study focuses on safety behavior of construction personnel.

Choudhry et al. [17] categorize safety performance measurement techniques into statistical measures, behavioral measures, periodic safety audits, and a balanced scorecard approach. The behavioral measures, safety audits, and balanced scorecard are time-consuming and not easy to measure by a questionnaire [38]. The statistical measures are used to measure construction personnel’s perceptions of safety performance on their own construction sites. The reliability and validity of the statistical measures can be guaranteed when the sample size is large enough. Because the primary data source of this study was a questionnaire survey of a random sample of local construction personnel, the research team decided to use the statistical measures to measure safety performance, i.e., construction personnel’s perceptions of their own safety behavior.

A unidimensional model of the safety behavior construct which focuses on compliance with safety rules and procedures is inappropriate [39]. Drawing on Borman and Motowidlo’s [40] work performance typology, Griffin and Neal [22] identify two categories of safety behavior: compliance and participation. Safety compliance concerns individuals carrying out core safety activities to maintain workplace safety, including adhering to safety rules and procedures and wearing personal protective equipment. Safety participation refers to behaviors that may not directly improve workplace safety but can help develop a safe environment, e.g., voluntarily participating in safety activities, and helping colleagues with safety issues. This dichotomy has implications for safety, because it highlights the need to devise different strategies to improve different aspects of safety behavior.

Thus far, a series of mediating relationships can be hypothesized as follows:

**Hypothesis H1.** *Safety-specific leader–member exchange mediates the relationship between transformational leadership and safety climate.*
**Hypothesis H2.** *Safety knowledge and motivation partially mediate the relationship between safety climate and safety behavior.*
**Hypothesis H3.** *Safety-specific leader–member exchange, safety climate, safety knowledge and safety motivation mediate the relationship between transformational leadership and safety behavior.*

**Figure 1:**
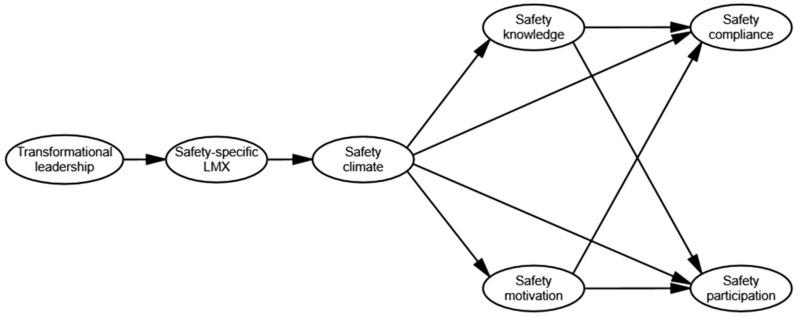
Hypothesized structural model.

These hypotheses are depicted in Figure 1.

## 3. Methods

### 3.1. Population and Sample

The target population was construction site personnel based in Hong Kong, who were grouped into eight sub-categories in three main categories. The category of contractor includes main contractors and subcontractors/workers, the category of consultant covers engineers, architects, and quantity surveyors, and the category of client includes those clients affiliated with the public, private, and quasi-government sectors. As the number of members in each category is unknown, the study constructed a sampling frame which consisted of members from local construction trade associations, professional bodies, government agencies, and property developers. The authors then selected a random sample of 2996 prospective respondents from the sampling frame and sent them hard-copy questionnaires. Five months later, the research team secured 292 valid responses. Table 1 shows the characteristics of respondents.

Due to the high turnover rate of local construction practitioners, 865 questionnaires were returned as non-deliverables. Given the non-deliverables, the survey response rate was 13.7%. As suggested by Armstrong and Overton [41], the research team carried out a time trend extrapolation test to check on non-response bias. Specifically, the research team labeled those valid responses received in the first month after questionnaire distribution as early responses, and the remaining as late responses. Then the researchers conducted chi-square tests to compare the early and late responses in terms of respondents’ individual attributes. As shown in Table 2, the researchers did not find significant differences between the early and late responses, and hence were assured that non-response bias was not an issue with the sample.

### 3.2. Measures

In a questionnaire survey, it is essential to secure prospective respondents’ cooperation and to make questionnaires self-contained and self-sufficient [42]. In order to achieve this, the research team performed the following tasks in designing the questionnaire: (1) using a straightforward rating format to elicit the degree to which respondents believe in the described phenomena, which can improve the scales’ reliability, validity and interpretability [43]; (2) using different response scales (i.e., six-point Likert scales to measure the constructs of transformational leadership and safety-specific LMX, and seven-point Likert scales to measure the constructs of safety climate, safety knowledge, safety motivation and safety behavior) to address common method variance, an issue which is associated with self-report questionnaires [44]; (3) assuring prospective respondents that their answers would be treated confidentially, which can increase accuracy and completeness of the information; and (4) consulting a group of researchers and practitioners with regard to the relevance of measurement scales, and conducting a pilot study with 18 poorly educated construction workers, to improve the scales’ content validity.

A research proposal and draft data collection instruments were submitted to and approved by the Human Research Ethics Committee for Non-clinical Faculties, the University of Hong Kong (HRECNF-HKU). The HRECNF-HKU approved on 6 October 2011, and assigned a reference number (i.e., EA011011) to the project. Measurement scales are presented in the following subsections.

#### 3.2.1. Transformational Leadership

The construct of transformational leadership refers to the behavioral style of a leader who encourages followers to transcend their own self-interests for the collective good. It was measured by six adapted items, which were the transformational leadership component of the Multifactor Leadership Questionnaire (MLQ, Form 5X). Using the MLQ (Form 1), Scholars developed a six-factor model of transactional and transformational leadership [45]. The six factors are charisma/inspirational, intellectual stimulation, individualized consideration, contingent reward, management-by-exception and *laissez-faire*. With a larger and more heterogeneous sample, Avolio et al. [45] confirmed the six-factor model of leadership using an updated version of MLQ Form 5X. The researchers adapted six items, with which Avolio et al. [45] described immediate supervisors’ transformational behaviors, to measure the construct of transformational leadership. The scale asked respondents to indicate the extent to which they agree with six statements. A sample statement read, “My immediate supervisor has my respect”. The scale items were anchored with the statement, “Please think about the working style of your immediate supervisor, to what extent do you believe in the following statements (1 = ‘to a small extent’, 6 = ‘to a large extent’)”.

#### 3.2.2. Safety-Specific Leader–Member Exchange

The construct features the interactions between employees and their immediate supervisors regarding safety issues. In measuring leader–member exchange, the LMX7, a 7-item LMX scale, has the soundest psychometric properties [46]. After adaption, the scale asked respondents to indicate to what extent they agree with seven statements in daily interactions with immediate supervisors on safety issues. A sample statement was, “My immediate supervisor understands my job problems and needs”. The seven statements shared the heading, “Please think about your daily interactions with your immediate supervisor about safety matters, to what extent do you feel (1 = ‘to a small extent’, 6 = ‘to a large extent’)”.

#### 3.2.3. Safety Climate

The construct reflects the extent to which construction personnel believe that safety is valued in their construction organization. It was measured by a 24-item scale, which was refined by [17,47,48,49] in a large scale safety climate questionnaire survey with construction personnel from a leading Hong Kong-based contractor. Hence, it was fitting for the Hong Kong construction practice [50]. Safety climate should be conceptualized as a higher order factor which is composed of more specific first-order factors. Those first-order factors should reflect perceptions of safety-related policies, procedures, and practices, while the higher order factor of safety climate is supposed to reflect the degree to which employees believe that safety is cherished in the organization [22]. Although large scale empirical studies on safety climate started in the early 1980s, it is premature to propose the factor structure of a higher order safety climate factor [22]. When assessing safety climate, the assessment purpose determines whether specific first-order factors or a global higher order factor is appropriate [22]. As this study was interested in assessing the overall impact of safety climate on safety behavior, it used the 24 items as a whole to measure the construct of safety climate. A sample item was, “Accidents and incidents which happen here are always reported”. The 24 items shared the same heading, “Please think about the project as a whole, to what extent do you believe in the following statements (1 = ‘to a small extent’, 7 = ‘to a large extent’)”.

#### 3.2.4. Safety Knowledge

The construct of safety knowledge reflects the extent to which construction personnel are knowledgeable about safety practices and procedures in the construction organization. It was assessed with a four-item scale developed by Neal et al. [20]. The scale asked respondents to rate the degree to which they agree with four statements. An example statement was, “I know how to perform work in a safe manner”. The four items were under the same heading, “Please indicate how much you know about the following things (1 = ‘to a small extent’, 7 = ‘to a large extent’)”.

#### 3.2.5. Safety Motivation

The construct of safety motivation reflects the degree to which construction personnel are inclined to perform tasks in a safe manner. It was also measured by a four-item scale developed by Neal et al. [20], asking respondents to indicate the extent to which they agree with four statements. An example statement was, “It is to my advantage to maintain or improve my personal safety”. The four items were under the same heading, “Please think about why you need to work safely on the project, to what extent do you believe in the following statements (1 = ‘to a small extent’, 7 = ‘to a large extent’)”.

#### 3.2.6. Safety Compliance and Safety Participation

The construct of safety compliance reflects the degree to which construction personnel are compliant with safety rules and procedures. It was measured by a four-item scale developed by Neal et al. [20], asking respondents to indicate the extent to which they agree with four statements. An example statement was, “I use all the necessary safety equipment to do my job”.

The construct of safety participation reflects the degree to which construction personnel participate in safety-related activities. It was measured by a four-item scale developed by Neal et al. [20], asking respondents to indicate the extent to which they agree with four statements. An example statement read, “I voluntarily carry out tasks or activities to improve safety”. Both four-item scales were under the same heading, “Please indicate to what extent do you carry out each of the following activities (1 = ‘to a small extent’, 7 = ‘to a large extent’)”.

#### 3.2.7. Demographic Information

The survey collected respondents’ demographic information, which can be used to check on non-response bias, as shown in Table 2. A complete questionnaire is available from the corresponding author on request.

### 3.3. Data Analysis

The research team carried out a questionnaire survey of a random sample of Hong Kong-based construction project personnel. Using the data, the authors tested the hypotheses with the structural equation modeling (SEM) technique. The reasons are twofold. First, the constructs in the model have to be approximately measured by multiple indicators, because they are difficult to measure directly. The ability of SEM to deal with poorly measured constructs makes it suitable for data analysis. Specifically, this study intends the indicators to be reflecting the focal construct they are measuring. For example, the item “it is to my advantage to maintain or improve my personal safety”, which is used to measure the construct of safety motivation, reflects the inclination of respondents to engage in safety behavior. Furthermore, individuals who have safety motivation are supposed to feel that maintaining or improving their personal safety is in their interest. Reflective indicators are interchangeable, and any single indicator can be deleted without undermining the focal construct [51]. Second, compared to standard multiple regression techniques, SEM can provide more accurate and reliable estimates of the relationships between constructs, because it takes into consideration measurement errors [51]. In an SEM model there are two types of constructs, i.e., exogenous and endogenous constructs. Exogenous constructs are determined by factors outside of the model, whereas endogenous constructs are dependent on exogenous constructs [51].

In general, the SEM method follows two steps [8]. First, it measures the reliability and validity of a set of indicators in representing the intended construct, and this is the measurement model assessment component of SEM. Reliability measures the degree to which an indicator or set of indicators is consistent in representing the intended construct, and validity concerns the degree to which an indicator or set of indicators is free from systematic errors in measuring the intended construct [51]. Specifying a complete measurement model requires (a) loading each item (i.e., reflective indicator) on the construct that it intends to measure; (b) correlating each pair of constructs; and (c) designating an error item for each item. Second, after obtaining reliable and valid measures of the constructs, the structural model estimates the relationships between constructs by assessing the significance of relationships between corresponding measures. This is the structural model assessment component of SEM. Transforming a measurement model into a structural model entails specifying relationships from exogenous construct(s) to endogenous construct(s) according to the conceptual framework. Each hypothesis is represented by a designated relationship. Hypotheses are supported when: (a) the structural model achieves acceptable goodness-of-fit; and (b) path estimates (i.e., standardized path coefficients) related to the hypotheses are statistically significant and in the hypothesized direction [51]. This study used the AMOS-24 software package [52] to execute the SEM procedures. In a typical AMOS path diagram output, an ellipse represents an unobservable construct, and a rectangle represents an observable indicator.

## 4. Results

### 4.1. Construct Reliability and Validity

Hair et al. [51] suggest that four indices, one incremental index (e.g., comparative fit index, CFI), one absolute index (e.g., root mean square error of approximation, RMSEA), the chi-square (χ^2^) value and the associated degrees of freedom (*df*), are sufficient to assess the overall goodness-of-fit of the measurement or structural model. This study follows this suggestion.

**Figure 2:**
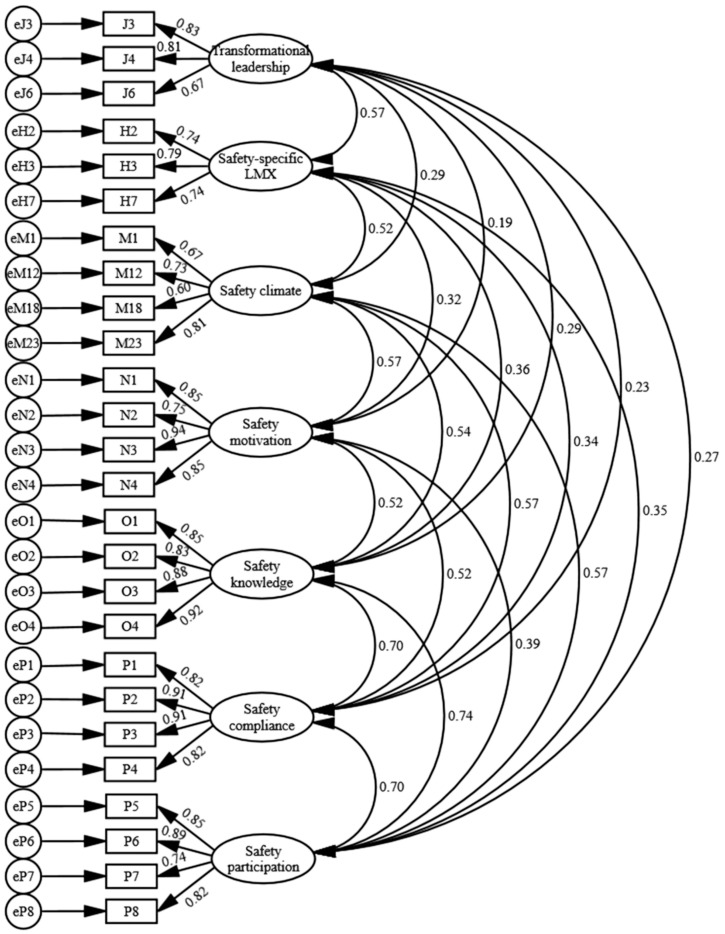
Final measurement model (χ^2^ = 526.65; *df* = 278; CFI = 0.953; RMSEA = 0.055). CFI: comparative fit index; RMSEA: root mean square error of approximation.

Many indicators are used to assess the reliability and validity of constructs. A frequently used construct reliability indicator is Cronbach’s alpha, with values of 0.60 to 0.70 deemed the lower limit of acceptability [51]. Two types of construct validity which are commonly reported are convergent and discriminant validity. Convergent validity assesses the degree to which a set of indicators representing a latent construct are highly correlated, and discriminant validity measures the extent to which one construct is distinct from others. The average variance extracted (AVE), a measure of convergent validity, is the average percentage of variance extracted among the indicators of a construct. A construct with AVE no less than 0.50 is deemed to possess convergent validity. Discriminant validity of two constructs is secured when both of their AVEs are greater than the square of the correlation between them [51]. Evidence of both convergent and discriminant validity can be provided in assessing the measurement model. After rounds of modification based on model diagnostics, the final measurement model with acceptable goodness-of-fit is shown in Figure 2. Table 3 shows means, standard deviations, Cronbach’s alpha, correlations, and AVEs of the latent constructs in the measurement model. All of the correlations were smaller than 0.85, suggesting the absence of multi-collinearity [51]. The Cronbach’s alphas were all greater than 0.70, providing evidence of construct reliability. AVEs of all the latent constructs were no less than 0.50, supporting convergent validity. Discriminant validity of all constructs was secured because the AVEs of any two constructs were greater than the squared bivariate correlation. Furthermore, the factor loadings of indicators to their corresponding construct were statistically significant at the 0.001 level (two-tailed), and all the factor loadings were no less than an acceptable value of 0.60.

### 4.2. Hypothesis Testing

The results of hypothesis testing are presented in Table 4. First, the hypothetical model (i.e., Model 1) shown in Figure 1 was estimated. Next, as transformational leadership has direct impact on safety behavior [53,54], a partially saturated model, with direct paths from transformational leadership to safety compliance and participation (Model 2), was estimated. The model with direct links from safety climate to safety behavior (i.e., Model 1) had better goodness-of-fit, because it yielded a lower chi-square value between those two models with the same degrees of freedom. Next, four partially saturated models, which link transformational leadership and safety climate to safety compliance and participation respectively, were estimated. Among these four models with equal degrees of freedom, the model with direct link from safety climate to safety participation (i.e., Model 6) had the best goodness-of-fit, because it produced the lowest chi square value. The final step compared Model 1 and Model 6, and the results are shown in Table 5. Table 5 suggests that the paths in the hypothetical model (i.e., Model 1) were sufficient to represent the covariation between safety climate and safety behavior.

**Figure 3:**
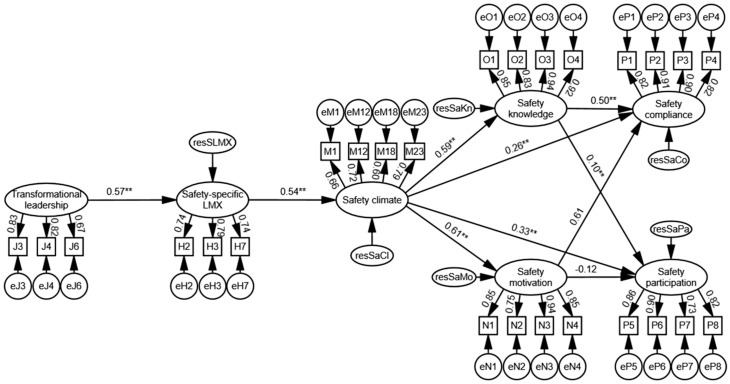
Final structural model (χ^2^ = 571.98; *df* = 289; CFI = 0.947; RMSEA = 0.058; ** *p* < 0.01).

Figure 3 shows the final structural model with acceptable goodness-of-fit, along with indicators and their standardized factor loadings, standardized path coefficients, and error terms for endogenous constructs. As mentioned earlier, if the standardized path coefficients are significant and in the hypothesized direction, then the corresponding hypothesis is supported. Hypothesis 1 predicted that safety-specific LMX mediates the relationship between transformational leadership and safety climate. This hypothesis was supported, because the two paths, transformational leadership → safety-specific leader–member exchange (standardized path coefficient = 0.57; *p* < 0.001) and safety-specific leader–member exchange → safety climate (standardized path coefficient = 0.54; *p* < 0.001), were significant. Hypothesis 2 suggested that safety knowledge and motivation partially mediate the relationship between safety climate and safety behavior. This hypothesis was partially supported, because only safety knowledge was found to partially mediate the relationship between safety climate and safety behavior. Because of the same reason, Hypothesis 3, which proposed that safety-specific LMX, safety climate, safety knowledge and safety motivation mediate the relationship between transformational leadership and safety behavior, was also partially supported.

## 5. Discussion

The actual effects of behavior-based safety interventions are often limited, because they merely focus on the immediate accident circumstances [55]. Contemporary construction accident causation models [6,7] adopt a systems perspective, and attribute incidents to a complex interaction between proximal factors (e.g., unsafe conditions and actions) and distal factors (e.g., management commitment and supervisor support). Occupational safety behavior research should also focus on both proximal person-related factors and distal situation-related factors [5]. Therefore, Christian et al. [5] conceptualized safety behavior as a complex interaction between proximal person-related factors and distal situation-related factors in a meta-analysis. However, relatively little research has examined this conceptualization in construction. This study modifies Christian’s et al. [5] model and takes leadership as an antecedent to safety climate. This treatment has both theoretical and practical reasons. In the literature involving the role of leadership in safety, essentially two perspectives are represented [56]. One investigates the impact of safety-specific leadership on safety outcomes [2,57,58,59], and the other investigates the relationship between general leadership constructs and safety outcomes [26,60,61]. Both perspectives indicate that leadership establishes a context where certain behavior is valued, rewarded, and expected [56]. Most effective supervisors are expected to display a supportive leadership style, proactively talk about safety with the workforce, and provide constructive feedback to workers regarding safety behavior [58,62,63]. The model modification also takes into consideration the sample’s cultural background, which has influence on their safety behavior. Hence, through examining the mechanisms by which transformational leadership impact safety behavior by the mediation of safety climate, safety knowledge and motivation, this study is expected to shed light on what management should do to translate transformational leadership into safety behavior before commencing the project.

The research team carried out a random questionnaire survey of construction personnel in Hong Kong, and applied the SEM technique in data analysis. The results revealed that construction personnel who feel more comfortable to discuss safety matters with their transformational supervisors (safety-specific LMX with transformational leadership) are more likely to feel that safety is valued in the workplace (safety climate), and more knowledgeable (safety knowledge) about carrying out tasks in a safe manner (safety compliance) and voluntarily helping to establish a safe work environment (safety participation). Contrary to expectations, safety climate predicted safety compliance and participation directly without influencing safety motivation. An explanation is that, the safety motivation scale used in the study did not capture all aspects of motivation relating to safety behavior. Four items of the safety motivation scale were “workplace health and safety is an important issue”, “it is to my advantage to maintain or improve my personal safety”, “maintaining safety at all times is important”, and “it is important to reduce the risk of accidents and incidents at work”. There was no extrinsic element in these four items, i.e., the scale merely reflected respondents’ intrinsic safety motivation. However, both of the safety compliance and participation scales reflected respondents’ performative safety behavior. Therefore, it is reasonable to suspect that from intrinsic safety motivation to performative safety behavior there are some mediator or moderator variables, which requires further examination.

The results have a number of implications for practice as well. First, a training program which aims to enhance transformational leadership and safety communication skills of supervisory staff may be a cost-effective way to create a positive safety climate. For example, interventions designed to create or improve a safety climate would be more effective if workers treat first-line supervisors as models, mentors and considerate friends, and feel free to raise safety issues. Second, safety climate has both direct and indirect impact on safety compliance and participation. Safety climate has direct influence on safety behavior, because individuals act based on their perceptions. An individual who perceives or feels that safety is valued in the workplace is very likely to act in a safe manner. Regarding indirect impact, the results highlighted the mediation role of safety knowledge. It suggests that even in a positive safety climate, employees need to be repeatedly reminded about safety on the job. This is relevant to the Hong Kong construction industry. For example, the Hong Kong construction sector is plagued with an aging workforce who are more likely to be influenced by entrenched working habits. In order to increase aged workers’ safety behavior, transformational first-line supervisors are expected to serve as a model in following safety practices, increase safety communication with aged workers, and repeatedly remind aged workers about safety.

This study, however, has limitations. The primary limitation is the use of a cross-sectional design, which makes it impossible to draw causal inferences from the findings. Another limitation is that the random sample was based in Hong Kong, and whether the findings apply in other cultural settings requires replication of this study.

Despite limitations above, this study makes both theoretical and practical contributions. In theory, it sheds lights on the mechanisms through which transformational leadership impacts on individual safety behavior. In practice, the authors suggest that future safety climate interventions be developed in combination with developing supervisory staff’s transformational leadership and safety communication skills, and improving construction personnel’s safety knowledge is essential for the combined effects of safety climate and leadership interventions to develop. The study was conducted at the individual level, and the model reflected a snapshot of the mechanisms to improve safety behavior. We, therefore, encourage researchers to study the mechanisms across different levels in a dynamic manner. As an anonymous reviewer indicates that an individual’s safety climate perceptions may be influenced by individual cultural values, we also encourage researchers to study the mechanisms across different cultural contexts.

## 6. Conclusions

Previous safety behavioral research conceptualized safety behavior as an interaction between proximal individual differences (safety knowledge and safety motivation) and distal contextual factors (leadership and safety climate). However, nearly no empirical research has examined this conceptualization in construction. Given the cultural background of the sample, this study slightly modifies this conceptualization and views transformational leadership as an antecedent of safety climate. Therefore, it aims to investigate the mechanisms by which transformational leadership translates into safety behavior. It depicts the mechanisms with a multiple mediator model, which later is estimated by the SEM technique using individual questionnaire responses from a random sample of construction personnel in Hong Kong. The results revealed that transformational leadership has a significant impact on safety climate through the mediation of safety-specific leader–member exchange, and safety climate in turn impacts safety behavior through safety knowledge.

## Figures and Tables

**Table 1:** Characteristics of respondents.

Characteristics	Frequency	Percentage (%)
Gender		
Female	22	7.5
Male	270	92.5
Age (years)		
20–30	19	6.5
31–40	51	17.5
41–50	104	35.6
>50	118	40.4
Marital status		
Single	49	16.8
Married	243	83.2
Education level		
Below primary	1	0.3
Primary	5	1.7
Secondary	24	8.2
Certificate/diploma	19	6.5
College or higher	243	83.3
Number of dependents		
0	21	7.2
1–2	131	44.9
3–4	125	42.8
5–6	11	3.8
>6	4	1.3
Industrial experience (years)		
<3	10	3.4
3–10	26	8.9
11–15	38	13.0
16–20	39	13.4
>20	179	61.3

**Table 2:** Chi-square tests to evaluate non-response bias.

Demographic Information	χ^2^ Value	Degrees of Freedom (*df*)	Significance (2-Tailed)
Gender	0.264	1	0.607
Age	2.471	3	0.481
Marital status	0.251	1	0.616
Number of dependents	2.434	4	0.657
Education level	7.565	4	0.109
Industrial experience	5.691	4	0.223

**Table 3:** Means, standard deviations, Cronbach’s alphas, average variances extracted, and correlation matrix.

Construct	Cronbach’s Alpha	Mean	Standard Deviation	Construct						
				SaCl	SaMo	SaKn	TFL	SLMX	SaCo	SaPa
SaCl	0.791	5.47	1.013	*0.50*	-	-	-	-	-	-
SaMo	0.903	6.36	0.779	0.509 **	*0.72*	-	-	-	-	-
SaKn	0.934	5.77	0.905	0.469 **	0.494 **	*0.78*	-	-	-	-
TFL	0.807	4.39	0.850	0.227 **	0.140 *	0.237 **	*0.60*	-	-	-
SLMX	0.797	4.65	0.810	0.429 **	0.287 **	0.308 **	0.478 **	*0.57*	-	-
SaCo	0.919	6.13	0.775	0.505 **	0.512 **	0.661 **	0.178 **	0.295 **	*0.75*	-
SaPa	0.891	5.54	1.123	0.478 **	0.386 **	0.690 **	0.215 **	0.301 **	0.655 *	*0.68*

(1) Abbreviations: SaCl = Safety climate; SaMo = Safety motivation; SaKn = Safety knowledge; TFL = Transformational leadership; SLMX = Safety-specific leader–member exchange; SaCo = Safety compliance; SaPa = Safety participation; (2) The constructs of transformational leadership and safety-specific leader–member exchange were measured with a six-point Likert scale; the constructs of safety climate, safety motivation, safety knowledge, safety compliance, and safety participation were measured with a seven-point Likert scale; (3) Correlations are below the diagonal. The italics on the diagonal are average variances extracted of the corresponding constructs; (4) ** *p* < 0.01; * *p* < 0.05.

**Table 4:** Comparison of alternative models.

Model No.	Model	χ^2^	*df*	CFI	RMSEA	Remark
1	Hypothesized model	571.98	289	0.947	0.058	Acceptable
2	Direct paths from transformational leadership to both safety compliance and participation	597.48	289	0.942	0.061	Acceptable
3	Direct path from transformational leadership to safety compliance	599.32	290	0.942	0.061	Acceptable
4	Direct path from transformational leadership to safety participation	597.76	290	0.942	0.060	Acceptable
5	Direct path from safety climate to safety compliance	591.52	290	0.943	0.060	Acceptable
6	Direct path from safety climate to safety participation	584.61	290	0.944	0.059	Acceptable

CFI = Comparative Fit Index; RMSEA = Root Mean Square Error of Approximation.

**Table 5:** Determination of the final structural model.

Model No.	Model	χ^2^	*df*	Δχ^2^	Δ*df*	Sig.	Remark
1	Direct paths from safety climate to both safety compliance and participation	571.98	289				
6	Direct path from safety climate to safety participation	584.61	290	12.63	1	<0.05	Model 1 preferred
